# Dyspnea affective response: comparing COPD patients with healthy volunteers and laboratory model with activities of daily living

**DOI:** 10.1186/1471-2466-13-27

**Published:** 2013-04-27

**Authors:** Carl R O’Donnell, Richard M Schwartzstein, Robert W Lansing, Tegan Guilfoyle, Daniel Elkin, Robert B Banzett

**Affiliations:** 1 Division of Pulmonary, Critical Care, and Sleep Medicine, Beth Israel Deaconess Medical Center, Boston, MA, 02215, USA; 2 Harvard Medical School Boston, Boston, MA, 02115, USA

**Keywords:** Dyspnea, Symptom assessment, COPD

## Abstract

**Background:**

Laboratory-induced dyspnea (breathing discomfort) in healthy subjects is widely used to study perceptual mechanisms, yet the relationship between laboratory-induced dyspnea in healthy volunteers and spontaneous dyspnea in patients with chronic lung disease is not well established. We compared affective responses to dyspnea 1) in COPD patients vs. healthy volunteers (HV) undergoing the same laboratory stimulus; 2) in COPD during laboratory dyspnea vs. during activities of daily living (ADL).

**Methods:**

We induced moderate and high dyspnea levels in 13 COPD patients and 12 HV by increasing end-tidal CO_2_ (P_ET_CO_2_) during restricted ventilation, evoking air hunger. We used the multidimensional dyspnea profile (MDP) to measure intensity of sensory qualities (e.g., air hunger (AH) and work/effort (W/E)) as well as immediate discomfort (A_1_) and secondary emotions (A_2_). Ten of the COPD subjects also completed the MDP outside the laboratory following dyspnea evoked by ADL.

**Results:**

COPD patients and HV reported similar levels of immediate discomfort relative to sensory intensity. COPD patients and HV reported anxiety and frustration during laboratory-induced dyspnea; variation among individuals far outweighed the small differences between subject groups. COPD patients reported similar intensities of sensory qualities, discomfort, and emotions during ADL vs. during moderate laboratory dyspnea. Patients with COPD described limiting ADL to avoid greater dyspnea.

**Conclusions:**

In this pilot study, we found no evidence that a history of COPD alters the affective response to laboratory-induced dyspnea, and no difference in affective response between dyspnea evoked by this laboratory model and dyspnea evoked by ADL.

## Background

How well do laboratory models contribute to our understanding of clinical dyspnea? Dyspnea is defined by the American Thoracic Society as “… a subjective experience of breathing discomfort that consists of qualitatively distinct sensations that vary in intensity. The experience … may induce secondary physiological and behavioral responses” [1]. This shares features with definitions of pain, which has been described as “an unpleasant sensory and emotional experience associated with actual or potential tissue damage, or described in terms of such damage” [2]. In the development of pain theory the affective dimension of pain has been divided into an initial stage (A1) of ‘immediate unpleasantness or discomfort’ and a later stage of emotional outcomes (A2) that may lead to behavioral outcomes [3-6]. The affective response to either pain or dyspnea can vary independently of sensory intensity [7,8] Thus, both dyspnea and pain are understood to have sensory and affective dimensions and, in pain theory, it is accepted that laboratory models have contributed to a better understanding of neurophysiological mechanisms and treatment [9]. (See Lansing et al., [10], for a more complete discussion of the multidimensional characteristics of pain and dyspnea.)

As is the case with pain models, a multidimensional perspective on dyspnea may also be useful for addressing question about the transferability of laboratory models to practice. A customary single-dimension measure of dyspnea, such as use of a modified Borg scale to rate sensory intensity (SI), may not adequately assess the differences seen between clinical and laboratory dyspnea. Although SI may predict affective response, the relationships among SI, A1 and A2 are likely to be influenced by factors such as sensory quality (SQ), pathophysiology, and environment. Thus, while laboratory models have been valuable in defining neural mechanisms of dyspnea, and in testing potential palliative interventions, they are prone to the criticism that clinical dyspnea is fundamentally different than laboratory dyspnea and that the experience of patients greatly alters their perceptual and emotional responses [11-14]. For example, laboratory interventions probably stimulate only a subset of the sensory receptors activated by pathophysiological processes and may give rise to different qualities of sensation. There are differences between the psychological environment of the laboratory and daily life: knowing that experimental risks are minimal, and that one can stop an experiment at any time, might reduce fear and anxiety compared to dyspnea associated with disease conditions that cannot be controlled.

In this study, we tested the extent to which one laboratory model of dyspnea (air hunger) evokes different affective responses between COPD patients and HV. We also assessed differences in affective responses of COPD patients to the lab dyspnea model and dyspnea associated with activities of daily living (ADL). Using a multidimensional measurement instrument that incorporates qualitative and affective measurements, we first compared dyspnea perceptions of Healthy and COPD volunteers in the laboratory [15]. We, then compared dyspnea perception in the laboratory with dyspnea perception in the daily lives of the same COPD volunteers. We tested three statistical null hypotheses:

H0_1 During experimentally induced dyspnea, the relationship between immediate affective response (unpleasantness, discomfort or A_1_) and the intensity of specific sensory qualities (SI) does not differ between healthy and COPD volunteers.

H0_2 During experimentally induced dyspnea, the relationship between immediate unpleasantness, discomfort and emotional affective response (A_2_ ) does not differ between healthy and COPD volunteers.

H0_3 In individuals with COPD, the relationship between immediate unpleasantness, discomfort and emotional affective response does not differ between dyspnea associated with activities of daily living (ADL) and experimentally induced dyspnea.

## Methods

### Subjects

Subjects at least 45 years old were recruited through advertisement and referral by pulmonary physicians. We screened subjects for self-reported cardiopulmonary or neurological conditions, and administered the Baseline Symptom Inventory (BSI-18) to identify subjects prone to panic disorder [16]. No subjects were excluded based on BSI results. In order to assure comparable baseline (pre-stimulus) measurement conditions between COPD and healthy volunteers, we selected COPD participants who reported dyspnea during exercise but not at rest. COPD diagnosis was confirmed by pulmonary function tests (PFTs) and compatible history and symptoms [17,18]. Healthy volunteers reported no history of physician-confirmed COPD, no use of respiratory medication, and no exercise limitation due to respiratory symptoms. Prior to each experimental day, subjects were asked about acute changes in health status such as respiratory infections, COPD exacerbation, and change in medication type or dose. The study protocol was approved by the Committee on Clinical Investigation of the Beth Israel Deaconess Medical Center, where all experiments were performed (Protocol # 2006P000468). All subjects gave written informed consent.

### Physiologic measurements

Subjects began each session by sitting comfortably and reading a standard text while end-tidal PCO_2_ (P_ET_CO_2_) was sampled in one nostril through an unobtrusive tube. During experiments, subjects breathed through a mouthpiece; tidal PCO_2_ and pressure at the airway opening were sampled in the common line, and we monitored pulse rate, arterial oxygen saturation and blood pressure throughout the experiment. Data were digitized and recorded for later analysis (PowerLab 16/30, AD Instruments, Colorado Springs, CO).

### Stimulus and protocol

Using a standardized script (see Additional file 1, text of standardized script), we instructed subjects on the details of the experimental protocol. On separate practice days, we familiarized subjects with the dyspnea experience to stabilize the relationship between stimulus and rating and to identify subjects who were unable to provide ratings reliably related to stimulus level [19]. Five unreliable raters (2 HV, 3 COPD) were excluded. These excluded subjects all had R^2^ value <0.49 (R < 0.7) for the regression of on-line discomfort ratings (BDVAS, defined below) on P_ET_CO_2_.

We employed a hypercapnic stimulus with minute ventilation constrained to 0.13 liters/min/kg (termed here 'standard ventilation', a level modestly greater than normal resting ventilation), by limiting flow to an inspiratory reservoir bag (see figure in Additional file 2 schematic of hypercapnic stimulus apparatus and on-line rating device). To control frequency and tidal volume, subjects breathed at 14 to 16 breaths per minute in time with an audible signal. The standard ventilation was achieved by all subjects with no evidence of dynamic hyperinflation. Stimulus strength, i.e., change in P_ET_CO_2_ above resting value, was varied 3 to 5 times in an arbitrary pattern to minimize subject expectation [20]. At each step, the goal was to achieve a constant P_ET_CO_2_ for 2 minutes. We used continuous on-line ratings to target experimental end-points of moderate and high levels of breathing discomfort (A1) (60% and 90% full scale [FS]).

Each test lasted approximately 15 minutes, and subjects completed a minimum of one test concluding at each of the two targeted end-points (moderate and high discomfort) The Multidimensional Dyspnea Profile (MDP) was completed immediately after each test, and subjects were instructed to focus on the final 30 to 60 seconds of the stimulus exposure (corresponding to the targeted end-point). Physiologic data associated with MDP ratings were averaged over the same 30 to 60 seconds.

### Measurement of dyspnea

During laboratory dyspnea challenge, subjects continuously rated ‘breathing discomfort (BD), or unpleasantness’ using an online ‘Visual Analog Scale’ (BDVAS) anchored with “No Discomfort” at 0 and “Stop Now” at 100% of full scale (%FS). ^a^ The experimenters immediately reduced the stimulus if the subject rated 100%. The BDVAS was intended as a continuous measure of A1 (immediate discomfort), and was used to target comparable levels of A1 among subjects prior to completion of the MDP (see Additional file 2).

Following each laboratory dyspnea challenge, and following periods of activity at home, subjects reported respiratory sensations and resulting emotional responses using the Multidimensional Dyspnea Profile (MDP) [8,15]. Respondents rated items on 10 point visual analog scales (VAS) related to: perceived overall sensory intensity (SI); immediate unpleasantness of the dyspnea experience (immediate affective response or A_1_); the intensity of specific sensory qualities (SQ) such as a sense of “air hunger” and “breathing work and effort” [21-23] and emotional responses such as fear, anxiety and frustration (secondary affective response, A_2_). Respondents also chose a single SQ that most aptly described the dyspnea sensation. (See Additional file 3 for a schematic representation of the MDP) The MDP, which can be completed in 2–4 minutes, is designed for both laboratory and clinical use, and can be administered immediately following an event or used for later recall. The construct validity, responsiveness and reliability of this instrument have been described [15].

### Protocol for dyspnea assessment during activities of daily living

After completing all laboratory experiments, COPD patients took home copies of the MDP to complete daily for 2 weeks following episodes of dyspnea associated with normal Activities of Daily Living (ADL). Patients also recorded the events that had provoked the reported dyspnea. MDP responses were averaged within subject to provide equal weighting, and were then compared to responses obtained from the same subjects following laboratory dyspnea.

### Statistical analyses

Descriptive statistics by subject type and stimulus intensity are reported as means and standard deviations. The statistical significance of between group (Healthy vs. COPD) item response differences was evaluated by the Mann–Whitney U test, while the Wilcoxon test was used to evaluate within group (by stimulus intensity) differences. Repeated measures ANOVA was used to assess group by stimulus intensity interactions. Additional analyses to assess internal consistency and test-retest reliability of the MDP in a laboratory setting are provided in Additional file 4 (MDP test-retest measures). Statistical analyses were performed using SPSS version 12.0.1.

## Results

### Subjects

Analyses were performed on 12 HV and 13 COPD patients whose characteristics are shown in Table 1. (See Additional file 5 for detailed subject data) We assessed the effect of stimulus strength using data from 11 HV and 12 COPD patients, for whom MDP responses were available at both moderate and high unpleasantness ratings. We assessed test-retest reliability of the MDP in nineteen subjects (12 COPD, 7 Healthy) who gave BDVAS ratings during the final minute of the laboratory stimulus on separate days that matched within 10% full scale (Additional file 4). Home MDP data were collected from 10 of the COPD patients who had been tested in the laboratory.

**Table 1:** Subject characteristics

	**N male (%)**	Age*	**Height (cm)**	**Weight (kg)**	**BMI (kg/m**^**2**^**)**	**BSI-18**	**Resting P**_**ET**_**CO**_**2 **_**(mmHg)**	**GOLD stages**	**FEV**_**1**_**/FVC**	**FEV**_**1, **_**(%Pred)**
COPD	7 (54%)	67.5	171.8	80.8	27.5	6.2	37.8	III 5	58.4	46.9
N = 13		(7.8^#^)	(8.8)	(22.1)	(7.9)	(6.1)	(1.8)	II 6	(22.1)	(15.8)
		(56–86^&^)						I 2		
Healthy	8 (67%)	54.8 (4.8)	172.2	77.3	25.9	2.1	40.0			
N = 12		(46–61)	(10.8)	(13.8)	(2.8)	(3.6)	(2.8)			

*P < 0.001 by Mann–Whitney U for difference between healthy and COPD subjects.

^#^(SD).

^&^(range).

### Laboratory challenge: COPD patients vs. HV

As would be expected from the similarity in underlying constructs, on-line BDVAS corresponded closely to subsequent MDP ratings of A_1_ (mean difference 2.5%FS; R^2^ = 0.704). There was no significant difference by subject type (p = 0.80 by two way ANOVA of subject type and stimulus level, (see Additional file 6 scatter plot of A_1_ vs. BDVAS by subject type).

**Figure 1:**
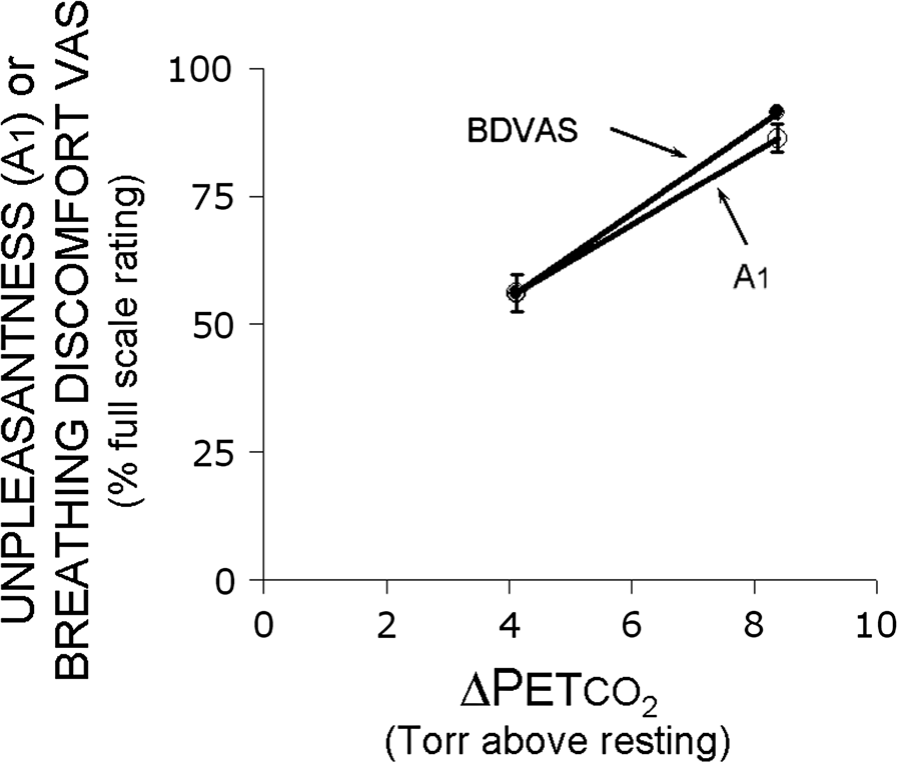
**Plot of BDVAS and A**_**1 **_**ratings during limited volume ventilation as a function of change in stimulus intensity (∆P**_**ET**_**CO**_**2**_**).** The BDVAS was used to continuously rate breathing discomfort throughout a trial lasting 10 to 15 min; the value shown in this graph is the mean BDVAS over the final 30 seconds of the trial. BDVAS scale ranged from zero to ‘stop now’, which indicated that the subject had reached his or her limit of tolerance. A_1_ was assessed immediately following the trial, and the subject was asked to focus on the last 30 seconds of the trial when responding. The A_1_ scale of unpleasantness ranged from ‘neutral’ to ‘unbearable’.

The stimulus levels (increase in P_ET_CO_2_ above resting) that evoked moderate (healthy 5.5 ± 3.7, COPD 2.8 ± 3.8) and high (healthy 10.5 ± 4.5, COPD 6.4 ± 7.5) ratings were not significantly different between subject groups (P = 0.18), and mean BDVAS and A_1_ were equivalent at both stimulus levels (Figure 1).

### Choice of sensory quality descriptors: COPD patients vs. HV

The set of descriptor phrases “I am not getting enough air, I feel hunger for air, or I am smothering” was chosen as the best descriptor by 52% of subjects following moderate, and 65% of subjects following high laboratory stimulus presentations. We term this descriptor group air hunger (AH). The descriptor phrase “I feel that my breathing requires work or effort” (WE) was chosen as the best descriptor by 35% of subjects following moderate and 22% of subjects following high laboratory stimulus presentations. All other descriptor phrases, such as “my chest or lungs feel tight”, or “I am breathing a lot” were chosen as best descriptor by only 13% of subjects following laboratory stimulus presentations. Best descriptor choice did not differ significantly between COPD and healthy subjects.

Despite discrimination of best descriptor choice at both stimulus levels, subjects provided roughly equal scale ratings of both sensory qualities; this apparent contradiction is discussed below. Following stimulus exposure at the moderate level, subjects rated AH at 46% full scale compared with 57% for WE (p = 0.035). Following exposure at the high level, corresponding ratings were 83% versus 86% (p = 0.223). Sensory quality ratings did not differ significantly by subject type.

### Unpleasantness and global sensory intensity responses: COPD patients vs. HV

**Figure 2:**
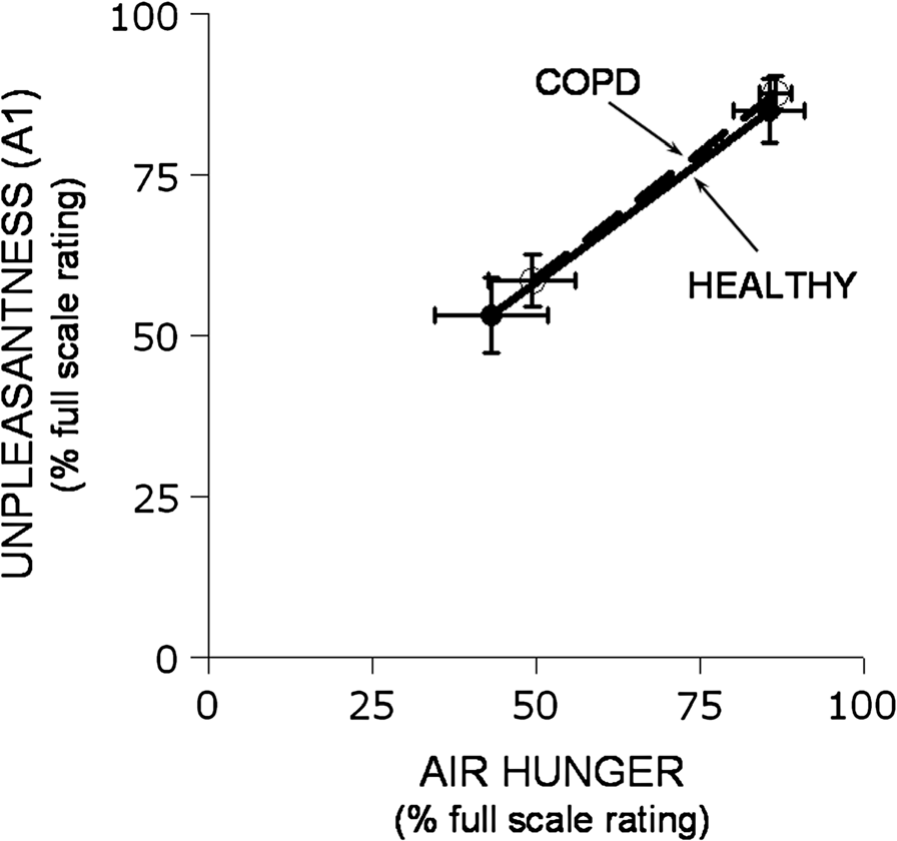
**Plot of ‘unpleasantness’ (A**_**1**_**) as a function of air hunger intensity for two groups of subjects, COPD patients (open circles, dashed line) and healthy controls (closed circles, solid line).** Both groups were exposed to moderate and high stimuli. There was no difference between the groups. The A_1_ scale of unpleasantness ranged from ‘neutral’ to ‘unbearable’; the air hunger scale ranged from zero (‘none’) to ‘as intense as I can imagine’.

A_1_ differed significantly between levels of perceived sensory intensity, (p < 0.001). We compared A_1_ ratings between groups at roughly matched levels of air hunger. There were no significant differences in mean ratings of A_1_ between healthy and COPD patients (by repeated measures ANOVA, p = 0.448). To account for small differences in sensory intensity within level, we plotted mean A_1_ versus mean ratings of AH by stimulus level and subject type (Figure 2).

### Emotional response to laboratory dyspnea: COPD patients vs. HV

**Figure 3:**
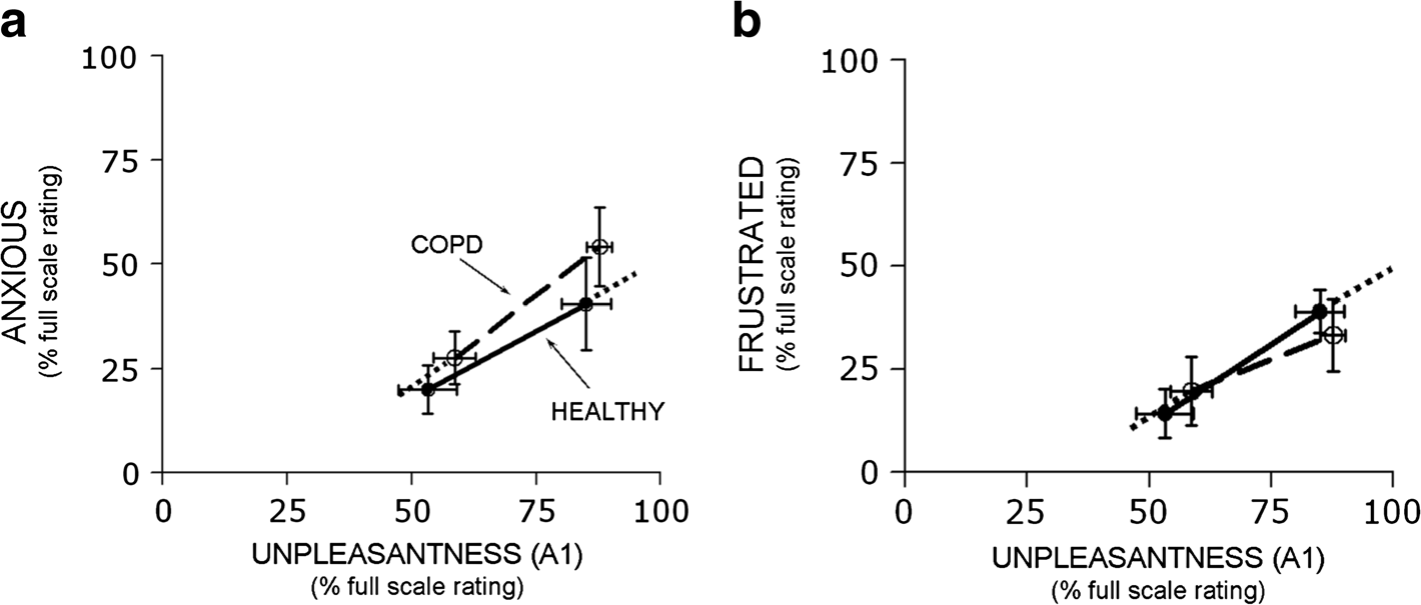
**Plot of anxiety rating (panel 3a) and frustration rating (panel 3b) vs. unpleasantness (A**_**1**_**) for healthy subjects (closed circles, solid line) and COPD patients (open circles, dashed line).** Ratings are for the last 30 seconds of moderate intensity and high intensity laboratory stimuli. Scale maxima were defined as ‘Unbearable’ for unpleasantness and ‘most I can imagine’ for emotion. Error bars reflect standard errors.

Both HV and COPD patients rated appreciable levels of “anxiety” and “frustration” related to laboratory exposure to the dyspnea challenge (Table 2). These emotions increased significantly at higher stimulus intensity (by repeated measures ANOVA, p < .001 for anxiety and p = .003 for frustration). However, there was no significant difference in emotional response between HV and COPD patients (Figure 3). For difference in rating by subject type (controlling for stimulus level), p = .321 and .952 for anxiety and frustration respectively. Both HV and COPD patients rated anger and depression less than 10% FS following stimulus exposure.

**Table 2:** **A**_**2 **_**responses of healthy volunteers and COPD patients to moderate and high laboratory stimuli**

**Stimulus level**	**Subject type**	**Anxious**	**Frustrated**	**Afraid**
Moderate	Healthy	20.0* (5.8)	14.2 (6.0)	5.9 (3.2)
	COPD	27.6 (6.3)	19.7 (5.2)	12.8 (5.1)
High	Healthy	40.5 (11.0)	39.0 (8.6)	18.6 (7.7)
	COPD	54.2 (9.4)	33.3 (8.7)	30.4 (10.0)

Ratings are given as % full scale ± se.

### Laboratory dyspnea vs. dyspnea of daily living (subset of 10 pts)

Ten COPD patients completed take-home dyspnea instruments. An average of 14 MDPs were completed by each subject following a separate instance of dyspnea over the course of two to three weeks. Fifty-one percent of 140 subject reports indicated that dyspnea episodes occurred while walking or climbing stairs, often when carrying groceries, laundry etc. Forty-eight percent of reports indicated that subjects curtailed the intensity or duration of ADL in order to limit dyspnea. Unfortunately the layout of the self-completed MDP used in this study failed to focus the subject’s attention on the requirement to choose the best descriptor, thus best-descriptor data were insufficient for analysis.

### Relationship of A_1_ to sensory intensity during ADL in COPD patients

**Figure 4:**
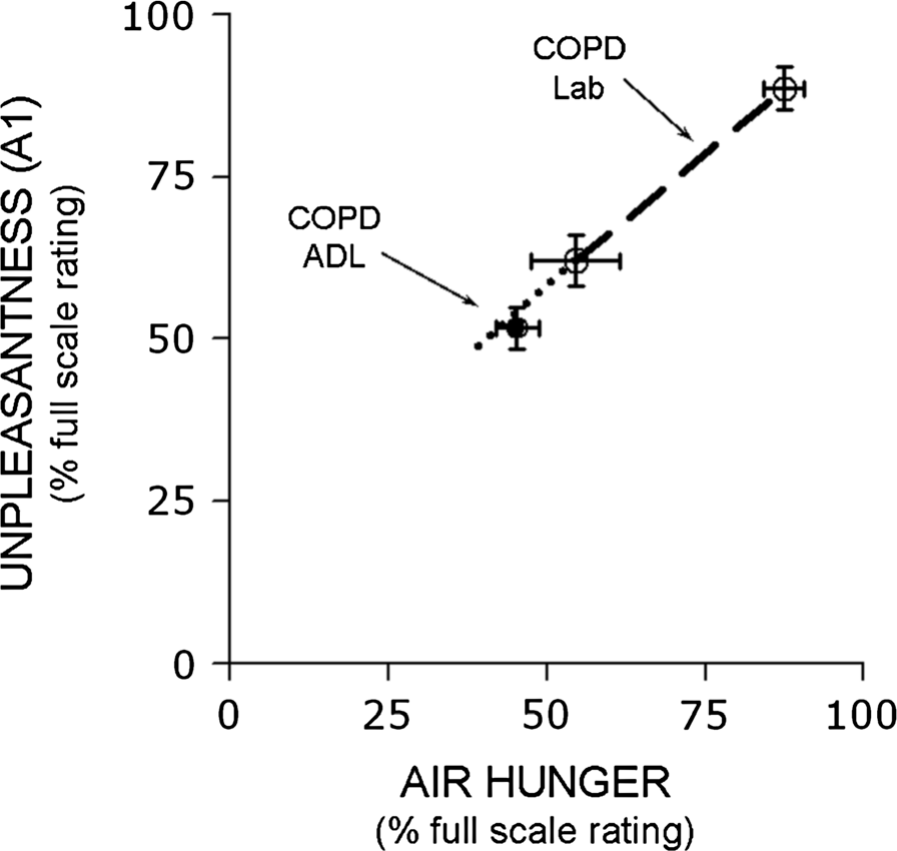
**Plot of Unpleasantness (A**_**1**_**) as a function of air hunger rating for the 10 COPD subjects who completed daily questionnaires at home.** Open circles connected by line represent dyspnea during moderate and high P_ET_CO_2_ during restricted ventilation in the laboratory; closed circle represents dyspnea experienced during activities of daily living, such as walking or climbing stairs. Scale maxima were defined as ‘Unbearable’ for unpleasantness and ‘as intense as I can imagine’ for air hunger. Error bars reflect standard errors.

The relationship between A_1_ and perceived air hunger was remarkably similar between ADL and the laboratory. Serendipitously, average A_1_ ratings associated with ADL were close to those earlier reported by the same subjects following the arbitrarily targeted moderate level of laboratory stimulus (Figure 4). This allows comparison of the other dimensions of dyspnea with reasonable confidence that non-linearity is not an issue.

The ratings of SQ during ADL were not significantly different than ratings obtained during the moderate level laboratory stimulus.

### Emotional response during ADL in COPD patients

**Figure 5:**
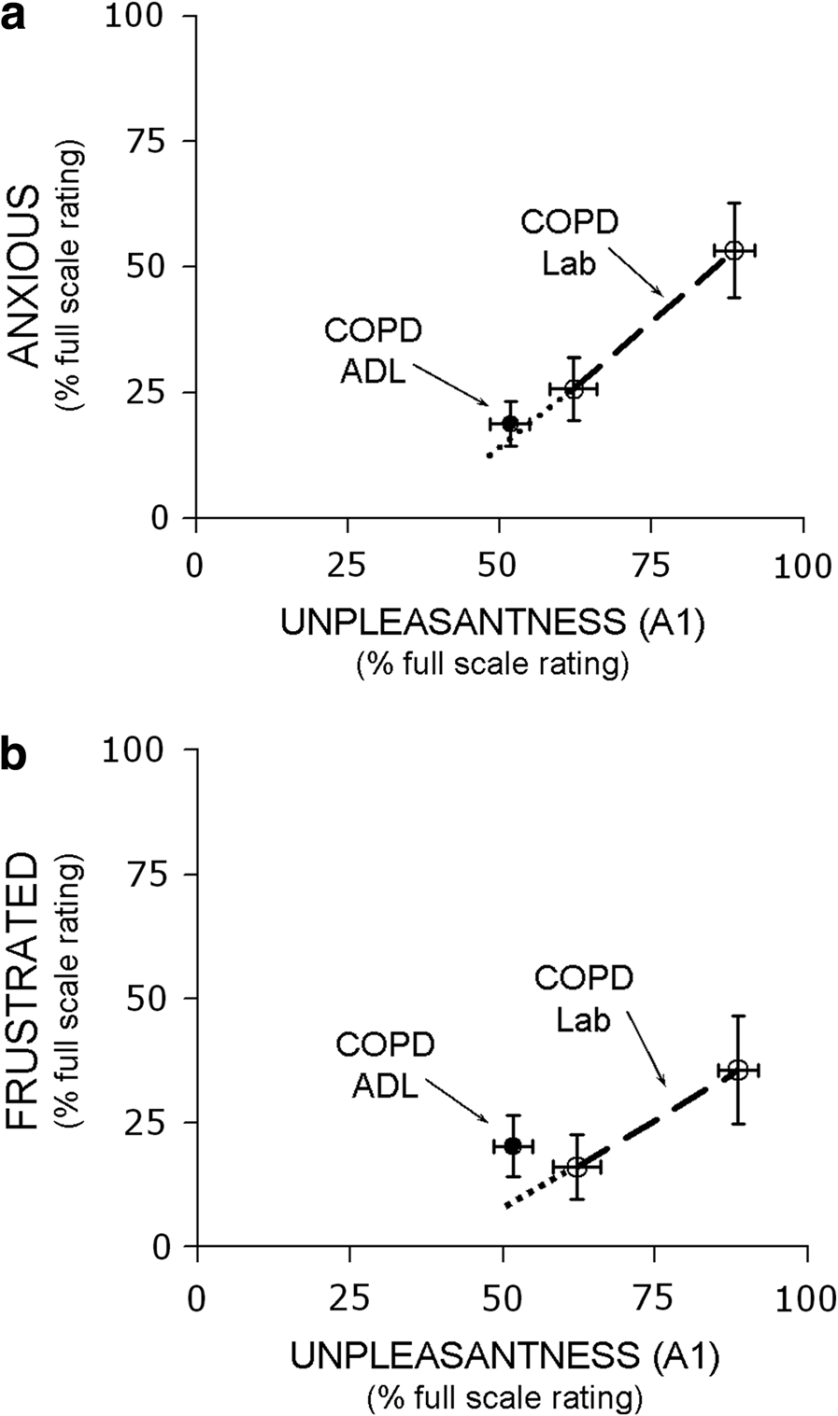
**Plot of anxiety (5a) and frustration (5b) ratings as a function of unpleasantness rating in COPD subjects during dyspnea caused by activities of daily living (ADL) and by a laboratory stimulus evoking air hunger.** Open circles connected by line represent dyspnea during moderate and high P_ET_CO_2_ during restricted ventilation in the laboratory; closed circle represents dyspnea experienced during activities of daily living, such as walking or climbing stairs. Scale maxima were defined as ‘Unbearable’ for unpleasantness and ‘most I can imagine’ for emotion. Error bars reflect standard errors.

The anxiety or frustration associated with respiratory discomfort was nearly the same whether the dyspnea was experienced during ADL at home or during moderate level laboratory stimulus (Figure 5). Subjects rated both anxiety (5a) and frustration (5b) at approximately 20% FS, about the same as during the moderate laboratory stimulus (p > 0.5).

## Discussion

### Summary conclusions

These are the first observations comparing multiple aspects of the dyspnea experience of HV and COPD patients following exposure to a laboratory dyspnea challenge that is physiologically equivalent in the two groups. Exercise has different physiologic implications for respiratory function in COPD, especially tidal volume limitation because of dynamic elevation of FRC [24,25], resulting in different qualities of dyspnea sensation and affective response [22]. Consequently, we selected a stimulus that evoked dyspnea without increased ventilation in an attempt to provoke the same sensation in both groups.

Our results are consistent with the three null hypotheses proposed: 1) COPD patients and HV report similar unpleasantness for a given intensity of sensation (AH and WE) in laboratory experiments; 2) COPD patients and HV reported similar emotional responses during laboratory dyspnea; 3) COPD patients reported similar frustration and anxiety with ADL as they did with laboratory dyspnea. Given the modest sample size, we consider below whether any important differences are likely to have gone undetected by this study (‘Type II error’).

### Do patients with chronic lung disease have a different affective response to dyspnea?

We showed that COPD patients and HV undergoing the same laboratory dyspnea experience report the same quality of sensation and the same immediate unpleasantness. This implies that long experience to clinical dyspnea has not greatly altered the immediate processing of respiratory discomfort. This is consistent with findings in competitive breath-hold divers whose perception of laboratory air hunger is not different from normal subjects [26].

We observed that the emotional component of dyspnea differed widely among individuals at both moderate and high strength laboratory stimulus levels (see graph in Additional file 7, interindividual variability of anxiety ratings (A2) following moderate and high level stimulus exposure). This type of individual difference in the emotional response to unpleasant stimuli has been termed affective style, and is likely an individual characteristic [27]. This affective style, captured by the MDP, may contribute to differences in clinical expression of symptoms and, ultimately, influence whether a particular individual seeks medical care for her symptom [15].

### In COPD patients, does the affective response to laboratory dyspnea challenge differ from the affective response to dyspnea in daily life?

COPD patients reported episodes of dyspnea during ADL that were similar in intensity and unpleasantness to our moderate strength laboratory dyspnea challenge. Ratings of anxiety, frustration and fear were in the lower third of the scale. Patients frequently reported that they limit physical activity in daily life to avoid high dyspnea levels, probably to avoid discomfort and adverse emotions. The relatively low A_2_ ratings may reflect a sense of control during daily activity. At higher levels of laboratory stimulus, ratings often exceeded 50%FS, but, probably because subjects were also in control of the maximal stimulus, anxiety ratings seldom reached 100%. This contrasts with emotional responses to dyspnea in acutely ill patients with inescapable dyspnea. For example, as noted by one patient after rating A_1_ at 75% FS and anxiety at 50%FS, “if I was alone and breathing like this I would be scared, would definitely feel more afraid [at home] not knowing how long it will last”. This is consistent with reports from patients seeking treatment in an emergency room [13].

### Limitations of this study

#### Sample size

We studied a relatively small number of subjects, thus the lack of statistically significant differences may reflect Type II errors. What is the likelihood that Type II errors could have obscured functionally important differences? The mean differences we report between affective responses by subject type (healthy vs. COPD) is virtually nil at moderate stimulus, and at most 14% FS at high stimulus for anxious (see Figure 3). Based on current data our best estimate is that anxiety in COPD patients during laboratory stimuli is very similar to healthy controls. However, because variance was large (coefficient of variation >60% for both HV and COPD patients), we can not state with confidence that anxiety in COPD is less than 20% FS different from controls following exposure to high intensity laboratory stimulus. Even this worst-case estimate would not be large enough to invalidate the use of the air hunger laboratory model for investigation of dyspnea. As described above, the large SD was mainly attributable to inter-, rather than intra-, individual variation of emotional responses in both subject groups. Repeat assessment in the laboratory (see Additional file 4) and clinical [15] setting demonstrate intraindividual consistency of affective ratings using the MDP. Thus, the effect of individual psychological characteristics seems to overwhelm the effect of chronic respiratory disease on the emotional response to dyspnea.

### Sample characteristics

COPD patients (67.5 ± 7.8) were older than healthy volunteers (54.8 ± 4.8). We found no reports of age associated differences in dyspnea perception during well-controlled stimulus exposure among adult subjects who differ in age by 10 to 15 years. Indirect evidence of age associated differences comes from studies of resistive load threshold detection among older asthmatics [28,29]. As we selected a stimulus not directly affected by mechanical load, we don’t think this evidence is relevant to interpretation of our results. To affirm this conclusion, we selected the 6 (50%) healthy volunteers (ages 56–61) and 8 (66%) COPD patients (ages 56–67) with overlapping age ranges. As in the larger groups, results for this subset were similar between subject types.

### Ability of MDP to discriminate sensory quality

There was a clear favorite choice of descriptor that best characterized the sensation during laboratory dyspnea, yet there was considerable overlap in the ratings of descriptors on a scale from 0–10. Our interpretation of this is that when subjects are presented with several parallel rating scales, many tend not to greatly separate ratings. On the other hand, when forced to make a choice about the most prominent sensation, the subject makes a judgment. As expected from results in earlier studies that exposed subjects to a similar dyspnea stimulus [8], air hunger was the best descriptor. In addition, high ratings of ‘work effort’ may reflect a combination of respiratory muscle effort and mental effort. Subjects presented with the supplementary descriptor “my breathing required concentration or mental effort” rated this item 62%FS and rated muscle work 56%FS.

### P_ET_CO_2_ measurement

The average changes from resting (∆P_ET_CO_2_) associated with the BDVAS ratings differed somewhat between COPD patients and HV, although the difference was not statistically significant. In COPD, P_ET_CO_2_ is not a reliable indicator of arterial blood gasses due to heterogeneous ventilation/perfusion ratio, the effect of which causes P_ET_CO_2_-PaCO_2_ difference to vary with tidal volume, T_E_, and PICO_2_-PaCO_2_ difference. Furthermore, ratings of SI for a given ∆PCO_2_ is known to vary widely among individuals [30]. For these reasons we chose to match stimulus strength based on on-line ratings, not on ∆PCO_2_. Because the range of ∆PCO_2_ was modest, approximately 2–10 mmHg, symptoms and signs attributable to elevated PaCO_2_, such as headache, elevated blood pressure and flushing, did not appear to affect discomfort ratings.

## Conclusions and implications

Our laboratory dyspnea model can produce sensory and affective responses in both HV and COPD patients similar to those experienced by COPD patients during exertion. Furthermore, similar sensory and affective responses are evoked in both healthy subjects and those with chronic disease. The laboratory intervention is capable of producing much stronger responses than moderately impaired COPD patients ordinarily allow themselves to experience during ADL, thus the laboratory intervention can be adjusted to match a wide range of clinical severity. We conclude that, despite very real differences between laboratory and clinical settings, laboratory results obtained by studying healthy individuals can be useful in examining mechanisms and testing treatments for patients with lung disease.

Our results carry implications for future dyspnea research. First, much dyspnea research is conducted on healthy individuals in laboratory settings; these data suggest that if the laboratory model is well chosen, such studies have relevance for our understanding of breathlessness associated with disease. Second, assessment of dyspnea has usually been limited to measurement within a single dimension such as intensity of a given, often non-specific, sensation. Our data argue that instruments such as the MDP can provide insight into affective as well as sensory experiences of dyspnea. The overall symptom burden of and functional limitation associated with clinical dyspnea may be heavily influenced by affective responses, and effective treatment of dyspnea, particularly in chronic disease, may depend upon manipulation of its affective components. Thus, the multidimensional assessment of both laboratory-evoked and naturally occurring dyspnea is an approach that can provide guidance for translating bench results to bedside application.

## Endnotes

^a^ Throughout the article the terms ‘unpleasantness’ and ‘discomfort’ are considered equivalent. While ‘Unpleasantness’ is more commonly used to describe the immediate affective response (A1) in multi multidimensional models of pain, ‘Discomfort’ is employed in the ATS dyspnea statement.

## Competing interests

Authors O’Donnell, Schwartzstein, Lansing, Gulifoyle, Elkin, and Banzett declare that they have no competing interests to report.

## Authors’ contributions

CRO participated in study design, study conduct, data collection and analysis, and manuscript preparation. RMS participated in study design, data analysis, and manuscript preparation. RWL participated in study design, data analysis, and manuscript preparation. TG participated in data management and analysis and in manuscript preparation. DE participated in study design, data collection, data management, and in manuscript preparation. RBB participated in study design, study conduct, data collection and analysis, and manuscript preparation. All authors have read and approved this manuscript in its final form.

## Pre-publication history

The pre-publication history for this paper can be accessed here:

http://www.biomedcentral.com/1471-2466/13/27/prepub

## Supplementary Material

Additional file 1 Text of standardized script. Click here for file

Additional file 2 Schematic of hypercapnic stimulus apparatus and on-line rating device. Click here for file

Additional file 3 Multidimensional Dyspnea Profile. Click here for file

Additional file 4 MDP Test-Retest Measures. Click here for file

Additional file 5 Subject Characteristics. Click here for file

Additional file 6 **Plot of MDP A1 ratings versus on-line BDVAS ratings showing close correspondence among both types of subjects.** (DOC 36 kb) Click here for file

Additional file 7 **Interindividual variability of anxiety ratings (A2) following moderate and high level stimulus exposure by subject type (Healthy and COPD).** Note that the range of ratings at matched A1 is similar between subject type. Click here for file

## References

[B1] ATS Committee on Dyspnea An official American thoracic society statement: update on the mechanisms, assessment, and management of dyspnea Am J Respir Crit Care Med 2012 185 435 452 10.1164/rccm.201111-2042ST 22336677 PMC5448624

[B2] International Association for the Study of Pain Task Force on Taxonomy Merskey H, Bogduk N Classification of chronic pain Classification of chronic pain 1994 2 Seattle, WA: IASP Press 210

[B3] Gracely R Affective dimensions of pain: how many and how measured? APS J 1992 1 243 247 10.1016/1058-9139(92)90056-I

[B4] Price DD Harkins SW The affective-motivational dimension of pain: a two stage model APS J 1992 1 229 239 10.1016/1058-9139(92)90054-G

[B5] Wade JB Dougherty LM Archer CR Price DD Assessing the stages of pain processing: a multivariate analytical approach Pain 1996 68 157 167 10.1016/S0304-3959(96)03162-4 9252011

[B6] Price DD Psychological and neural mechanisms of the affective dimension of pain Science 2000 288 1769 1772 10.1126/science.288.5472.1769 10846154

[B7] Gracely RH Dubner R McGrath PA Narcotic analgesia: fentanyl reduces the intensity but not the unpleasantness of painful tooth pulp sensations Science 1979 203 1261 1263 10.1126/science.424753 424753

[B8] Banzett RB Pedersen SH Schwartzstein RM Lansing RW The affective dimension of laboratory dyspnea: air hunger is more unpleasant than work/effort Am J Respir Crit Care Med 2008 177 1384 1390 10.1164/rccm.200711-1675OC 18369200 PMC2427058

[B9] Edwards RR Sarlani E Wesselmann U Fillingim RB Quantitative assessment of experimental pain perception: multiple domains of clinical relevance Pain 2005 114 315 319 10.1016/j.pain.2005.01.007 15777856

[B10] Lansing RW Gracely RH Banzett RB The multiple dimensions of dyspnea: review and hypotheses Respir Physiol Neurobiol 2009 167 53 60 10.1016/j.resp.2008.07.012 18706531 PMC2763422

[B11] Banzett RB Lansing RW Brown R High-level quadriplegics perceive lung volume change J Appl Physiol 1987 62 567 573 3558216 10.1152/jappl.1987.62.2.567

[B12] Banzett RB Lansing RW Brown R Topulos GP Tager D Steele SM Londono B Loring SH Reid MB Adams L ‘Air hunger’ from increased PCO_2_ persists after complete neuromuscular block in humans Respir Physiol 1990 81 1 17 10.1016/0034-5687(90)90065-7 2120757

[B13] Banzett RB Adams L O’Donnell CR Gilman SA Lansing RW Schwartzstein RM Using laboratory models to test treatment: morphine reduces dyspnea and hypercapnic ventilatory response Am J Respir Crit Care Med 2011 184 920 927 10.1164/rccm.201101-0005OC 21778294 PMC3208656

[B14] Banzett RB O’Donnell CR Guilfoyle T Lansing R Schwartzstein RM Is the experience of laboratory dyspnea different from wild-type dyspnea in COPD patients? [abstract] Am J Respir Crit Care Med 2011 183 A5810

[B15] Meek PM Banzett RB Parshall MB Gracely RH Schwartzstein RM Lansing RW Reliability and validity of the multidimensional dyspnea profile (MDP) Chest 2012 141 6 1546 1553 10.1378/chest.11-1087 22267681 PMC3367480

[B16] Derogatis LR Melisaratos N The brief symptom inventory: an introductory report Psychol Med 1983 13 595 605 10.1017/S0033291700048017 6622612

[B17] Global strategy for the diagnosis, management, and prevention of chronic obstructive pulmonary disease[http://www.goldcopd.org/guidelines-global-strategy-for-diagnosis-management.html]

[B18] Miller MR Crapo R Hankinson J Brussassco V Burgos F Casaburi R Coates A Enright P van der Grinten CP Gustafsson P Jensen R Johnson DC MacIntyre N McKay R Navajas D Pedersen OF Pellegrino R Viegi G Wanger J ATS/ERS Task Force General considerations for lung function testing Eur Respir J 2005 26 153 161 10.1183/09031936.05.00034505 15994402

[B19] Revelette WR Zechman FW Jr Parker DE Wiley RL Effect of background loading on perception of inspiratory loads J Appl Physiol 1984 56 404 410 6706751 10.1152/jappl.1984.56.2.404

[B20] Bloch-Salisbury E Shea SA Brown R Evans K Banzett RB Air hunger induced by acute increase in PCO2, adapts to chronic elevation of PCO, in ventilated humans J App Physiol 1996 81 Z 949 956 10.1152/jappl.1996.81.2.949 8872667

[B21] Simon PM Schwartzstein RM Weiss JW Distinguishable types of dyspnea in patients with shortness of breath Am Rev Respir Dis 1990 142 1009 1014 10.1164/ajrccm/142.5.1009 2240820

[B22] Schwartzstein RM Simon PM Weiss JW Breathlessness induced by dissociation between ventilation and chemical drive Am Rev Respir Dis 1989 139 1231 1237 10.1164/ajrccm/139.5.1231 2523682

[B23] Schwartzstein R Mahler D The language of dyspnea Dyspnea 1998 London: Marcel Dekker 35 62

[B24] O’Donnell DE Hyperinflation, dyspnea, and exercise tolerance in chronic obstructive pulmonary disease Proc Am Thor Soc 2006 3 180 184 10.1513/pats.200508-093DO 16565429

[B25] O’Donnell DE Banzett RB Carrieri-Kohlman V Casaburi R Davenport PW Gandevia SC Gelb AF Mahler DA Webb KA Pathophysiology of dyspnea in chronic obstructive pulmonary disease: a roundtable Proc Am Thorac Soc 2007 4 145 169 10.1513/pats.200611-159CC 17494725

[B26] Binks AP Vovk A Ferrigno M Banzett RB The air hunger response of four elite breath-hold divers Respir Physiol Neurobiol 2007 159 2 171 177 10.1016/j.resp.2007.06.014 17702673 PMC2225349

[B27] Krone HW Davidson RJ, Scherer KR, Goldsmith HH Individual differences in emotional reactions and coping Handbook of affective sciences 2003 Oxford: Oxford University Press 698 728

[B28] Allen SC Vassallo M Khattab A The threshold for sensing airflow resistance during tidal breathing rises in old age: implications for elderly patients with obstructive airways diseases Age Ageing 2009 38 548 552 10.1093/ageing/afp110 19589812

[B29] Allen SC Khattab A The airflow resistance sensing threshold during tidal breathing rises in old age in patients with asthma Age Ageing 2012 41 557 560 10.1093/ageing/afs041 22427506

[B30] Banzett RB Lansing RW Evans KC Shea SA Stimulus–response characteristics of CO2-induced air hunger in normal subjects Resp Physiol 1996 103 19 31 10.1016/0034-5687(95)00050-X 8822220

