# Supplementary material for: Dyspnea affective response: comparing COPD patients with healthy volunteers and laboratory model with activities of daily living
Source: BMC Pulm Med. 2013 Apr 27;13:27. doi: 10.1186/1471-2466-13-27 (PMC3663820; doi:10.1186/1471-2466-13-27)
Supplement: Additional file 1 — Text of standardized script. [file 1471-2466-13-27-S1.doc]

**Additional File 1**

Standard Lab Script

Subjects were instructed regarding the performance of the hypopnea stimulus task as follows:

Sometimes you will feel that your breath stops and you will not be able to take in more than a certain amount of air with each breath. When you feel the limit, it is best to relax, breathe out, and wait for more air to come into the bag*. Don’t try to suck in too hard, it won’t get you any more air. You will breathe in time with a sound signal that makes a periodic ‘whoop’ sound. You should breathe in during the whoop, and relax and breathe out during the silent period.

*Referring to a reservoir bag into which flow was supplied at 0.13 L/Min/Kg. Because rate was fixed by instructing the subject to breathe in time with a sound signal, this effectively clamped tidal volume.
