# Supplementary material for: Dyspnea affective response: comparing COPD patients with healthy volunteers and laboratory model with activities of daily living
Source: BMC Pulm Med. 2013 Apr 27;13:27. doi: 10.1186/1471-2466-13-27 (PMC3663820; doi:10.1186/1471-2466-13-27)
Supplement: Additional file 2 — Schematic of hypercapnic stimulus apparatus and on-line rating device. [file 1471-2466-13-27-S2.doc]

**Additional File 2**

Page 1 of 2

Schematic of Experimental Apparatus

During Dyspnea Challenge administration, subjects breathed through a mouthpiece connected to a non-rebreathing valve system via a viral filter/re-humidifier (Airlife HEPA, Cardinal Health, McGaw Park IL); inspired gas was supplied from a 5-liter rubber anesthesia bag and expired gas exited to the room via a one-way valve. Subjects inspired through a one way valve connected to the anesthesia bag. Gas was supplied to the bag via a high output impedance source (a needle valve with upstream pressure at 50psi), thus the subject could not inspire more gas than supplied by the source. Flow could be finely adjusted and was metered. Inspired PCO_2_ was adjusted by the operator to target desired levels of PETCO_2_. Flow rate was adjusted to meet the needs of the stimulus. Inspired oxygen concentration remained high, at 30%. Gas entering the bag was mixed from sources containing 30% O_2_ and either zero CO_2_ or 10% CO_2_.

Ventilation was limited to 0.13 liters/min/kg based on previous observations using a similar dyspnea challenge that indicated subjects were unlikely to exceed this ventilation while breathing on a mouthpiece with nose clips and no added inspired CO_2_. Measurements performed on the first seven subjects in the current experiment (5 healthy, 2 COPD) found that, on average, spontaneous ventilation while breathing room air on the apparatus was within .04 liters of experimental baseline ventilation.


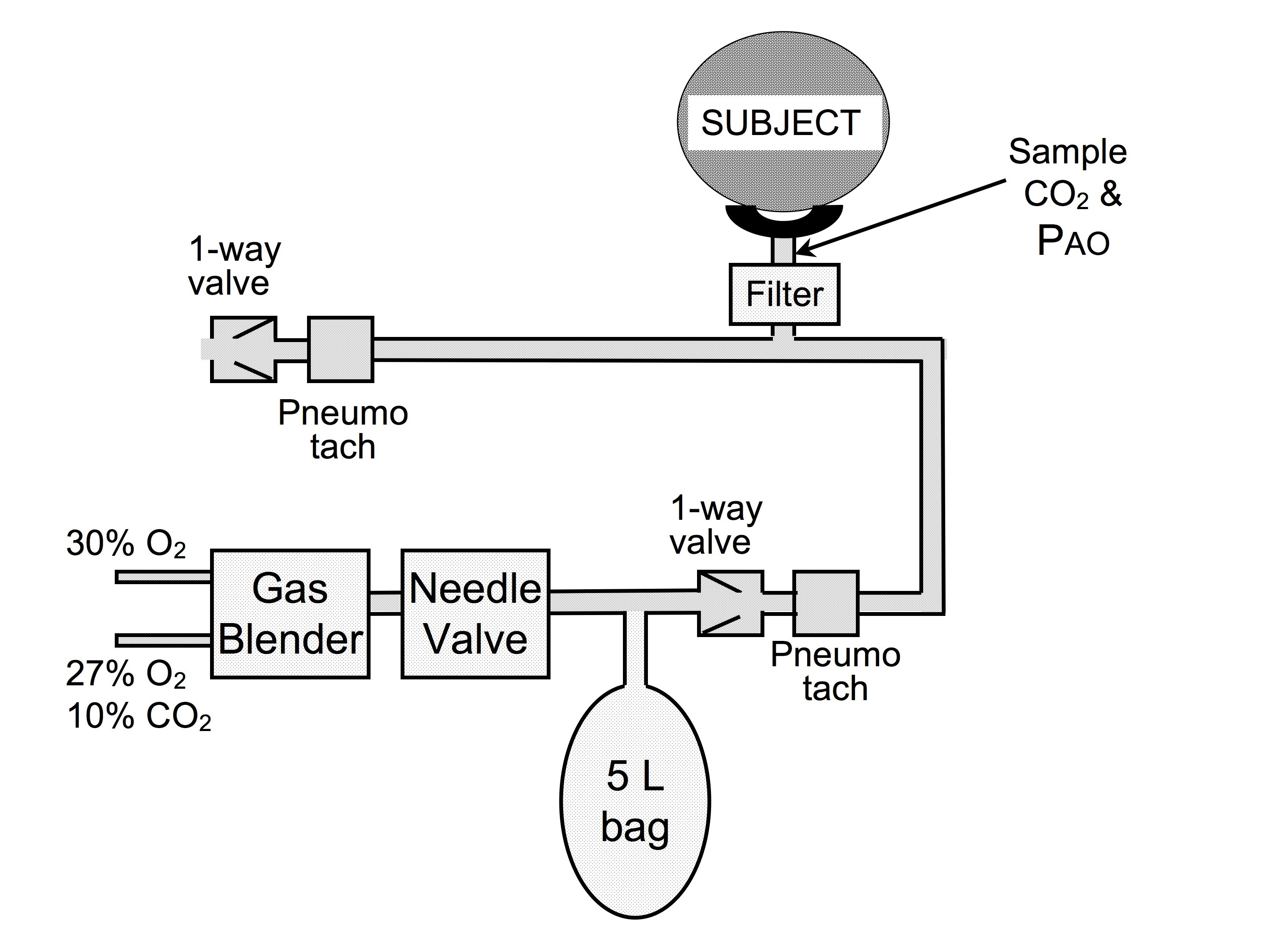


Page 2 of 2

No

Discomfort

Stop Now

Moderate

Discomfort

Mild

Discomfort

**Breathing**

**Discomfort**

10

9

8

7

6

5

4

3

2

1

0

No

Discomfort

Stop Now

Moderate

Discomfort

Mild

Discomfort

**Breathing**

**Discomfort**

10

9

8

7

6

5

4

3

2

1

0

Representation of on-line BDVAS rating device

showing moderate and high discomfort ratings

Throughout stimulus presentation, subjects used a hand-turned knob to illuminate

0-20 LEDs displayed vertically adjacent to a 10 point scale and verbal descriptors. They were instructed to rate “breathing discomfort or unpleasantness”, and to adjust their rating (up or down) whenever they perceived a change in discomfort.
