# Supplementary material for: Dyspnea affective response: comparing COPD patients with healthy volunteers and laboratory model with activities of daily living
Source: BMC Pulm Med. 2013 Apr 27;13:27. doi: 10.1186/1471-2466-13-27 (PMC3663820; doi:10.1186/1471-2466-13-27)
Supplement: Additional file 3 — Multidimensional Dyspnea Profile. [file 1471-2466-13-27-S3.doc]

**Additional File 3, Multidimensional Dyspnea Profile**

**Page 1 of 2**

**NOTE:**

**This representation of the MDP does not reflect the version in current use. Investigators interested in using the MDP are encouraged to contact the authors at http://shortofbreath.org/**

**Please focus on how your breathing felt during the final 30 seconds on the mouthpiece**.

**Sensory Intensity (SI)**

Use this scale to rate the intensity or strength of your breathing sensations.

| **0** | **1** | **2** | | **3** | **4** | | **5** | **6** | | **7** | **8** | | **9** | **10** |
| --- | --- | --- | --- | --- | --- | --- | --- | --- | --- | --- | --- | --- | --- | --- |
| No Sensation | | | Slight  Sensation | | | Moderate Sensation | | |  | | | Maximum Sensation | | |

**Immediate Unpleasantness (A1)**

Use this scale to rate the unpleasantness (or discomfort) of your breathing sensations, how good or bad your breathing feels [felt].

|  | **0** | | **1** | **2** | | **3** | **4** | | **5** | **6** | | **7** | **8** | **9** | | **10** |  |
| --- | --- | --- | --- | --- | --- | --- | --- | --- | --- | --- | --- | --- | --- | --- | --- | --- | --- |
|  | | Neutral | | | Unpleasant | | | Annoying | | | Distressing | | | | Unbearable | | |

Please go on to the next page

**Page 2 of 2**

**Sensory Qualities (SQ):**

Rate the Intensity of your Breathing Sensations.

None As intense as

I can Imagine

My breathing requires work or effort 0 1 2 3 4 5 6 7 8 9 10

I am smothering, I feel a hunger for air 0 1 2 3 4 5 6 7 8 9 10

My chest and lungs feel tight, constricted 0 1 2 3 4 5 6 7 8 9 10

I am breathing a lot 0 1 2 3 4 5 6 7 8 9 10

(breathing rapidly, deeply or heavily)

**Emotional Response (A2)**

Rate the Intensity of how your Breathing makes your Feel.

None As intense as

I can Imagine

Depressed 0 1 2 3 4 5 6 7 8 9 10

Anxious 0 1 2 3 4 5 6 7 8 9 10

Frustrated 0 1 2 3 4 5 6 7 8 9 10

Angry 0 1 2 3 4 5 6 7 8 9 10

Afraid 0 1 2 3 4 5 6 7 8 9 10
