# Supplementary material for: Dyspnea affective response: comparing COPD patients with healthy volunteers and laboratory model with activities of daily living
Source: BMC Pulm Med. 2013 Apr 27;13:27. doi: 10.1186/1471-2466-13-27 (PMC3663820; doi:10.1186/1471-2466-13-27)
Supplement: Additional file 4 — MDP Test-Retest Measures. [file 1471-2466-13-27-S4.doc]

**Additional File 4**

MDP Test-Retest Measures

Among the 19 subjects with matched BDVAS ratings during repeated experimental exposures there was, on average, good test-retest agreement of SI, A1 , SQ and A2 . The PETCO_2_ exposures that evoked these ratings were the same, on average, during repeat testing.

|  | On-Line VAS* | SI* | A1 * | WE* | AH* | Anxiety* | P_ET_CO_2_^#^ |
| --- | --- | --- | --- | --- | --- | --- | --- |
| Difference | 0.9% | 2.9% | 3.7% | 3.7% | 3.2% | 6.8% | -0.3 |
| SD | 5.7% | 19.0% | 18.7% | 23.4% | 31.0% | 33.8% | 5.4 |

* Column numbers represent Time 1 - Time 2 differences in % full scale.

# Column numbers represent Time 1 - Time 2 differences in mmHg

Scale Consistency and Reliability

To assess internal consistency (within subject item correlations) of ratings on the four primary SQ (AH, WE, tight/constricted, and rapid or deep) and five A2 items, we calculated Cronbach's alpha (α). The SQ items showed the best internal scale reliability (α = .81) when all four items were included. In contrast, the A2 items demonstrating best internal scale reliability were anxiety, frustration, and fear (α = .83), while addition of anger and/or depression decreased α. To assess intraindividual test-retest reliability of the MDP when applied in a laboratory setting, we computed summative SQ (4 items) and A2 (3 items) scale scores. These scores, along with corresponding values of SI and A1 , were used to compute repeated measures ANOVA comparing individuals Reponses to stimulus exposures with equivalent BDVAS ratings (+/- 10% full scale) and PETCO_2_ (mean difference 0.3 mmHg) that occurred on separate testing days.

With repeated exposures to the same dyspnea stimulus, resulting in equivalent on-line VAS ratings, SI, A1 , and both SQ and A2 scale scores were adequately reproducible, especially considering the relatively small sample size.

ICC estimates for MDP components

Measures ICC

Stimulus Intensity (SI) .612

Immediate Unpleasantness (A1 ) .575

SQ Scale (4 items) .721

Emotional Response (A2 ) (3 items) .628
