# Supplementary material for: Dyspnea affective response: comparing COPD patients with healthy volunteers and laboratory model with activities of daily living
Source: BMC Pulm Med. 2013 Apr 27;13:27. doi: 10.1186/1471-2466-13-27 (PMC3663820; doi:10.1186/1471-2466-13-27)
Supplement: Additional file 5 — Subject Characteristics. [file 1471-2466-13-27-S5.doc]

**Additional File 5**

Subject Characteristics

| Subject | Sex | Age | Height | Weight | BMI | Resting PETCO_2_ |  |
| --- | --- | --- | --- | --- | --- | --- | --- |
| **Healthy** |  |  |  |  |  |  |  |
| HV1 | M | 59 | 167.6 | 81.8 | 29.1 | 40 |  |
| HV2 | M | 61 | 177.8 | 87.3 | 27.6 | 41 |  |
| HV3 | F | 48 | 160.0 | 59.1 | 23.1 | 39 |  |
| HV4 | M | 52 | 181.6 | 80.0 | 24.3 | 42 |  |
| HV5 | M | 59 | 177.8 | 72.7 | 23.0 | 36 |  |
| HV6 | M | 56 | 176.5 | 93.6 | 30.0 | 38 |  |
| HV7 | F | 53 | 162.6 | 72.7 | 27.5 | 40 |  |
| HV8 | F | 53 | 162.6 | 56.4 | 21.3 | 44 |  |
| HV9 | M | 58 | 174.0 | 88.6 | 29.3 | 35 |  |
| HV10 | M | 60 | 177.8 | 80.5 | 25.4 | 43 |  |
| HV11 | F | 53 | 154.9 | 58.2 | 24.2 | 43 |  |
| HV12 | M | 46 | 193.0 | 97.3 | 26.1 | 42 |  |
|  |  |  |  |  |  |  |  |
| **COPD** |  |  |  |  |  |  | GOLD Stage |
| COPD1 | M | 65 | 182.9 | 103.6 | 31.0 | 36 | 3 |
| COPD2 | M | 86 | 182.9 | 73.2 | 21.9 | 38 | 3 |
| COPD3 | M | 72 | 182.9 | 70.9 | 21.2 | 39 | 3 |
| COPD4 | M | 69 | 168.9 | 50.9 | 17.8 | 40 | 3 |
| COPD5 | F | 56 | 167.6 | 43.2 | 15.4 | 39 | 3 |
| COPD6 | M | 71 | 182.9 | 97.7 | 29.2 | 38 | 2 |
| COPD7 | F | 61 | 166.4 | 80.9 | 29.2 | 40 | 2 |
| COPD8 | F | 65 | 160.0 | 57.3 | 22.4 | 39 | 2 |
| COPD9 | F | 61 | 162.6 | 93.6 | 35.4 | 38 | 1 |
| COPD10 | M | 77 | 172.7 | 97.3 | 32.6 | 36 | 2 |
| COPD11 | F | 67 | 165.1 | 120.5 | 44.2 | 38 | 1 |
| COPD12 | M | 67 | 176.5 | 77.3 | 24.8 | 35 | 2 |
| COPD13 | F | 61 | 162.6 | 84.1 | 31.8 | 39 | 2 |
