# Supplementary material for: Dyspnea affective response: comparing COPD patients with healthy volunteers and laboratory model with activities of daily living
Source: BMC Pulm Med. 2013 Apr 27;13:27. doi: 10.1186/1471-2466-13-27 (PMC3663820; doi:10.1186/1471-2466-13-27)
Supplement: Additional file 6 — Plot of MDP A1 ratings versus on-line BDVAS ratings showing close correspondence among both types of subjects. (DOC 36 kb) [file 1471-2466-13-27-S6.doc]

**Additional File 6**

Plot of MDP A1 ratings versus on-line BDVAS ratings showing close correspondence among both types of subjects.
