# Supplementary material for: Dyspnea affective response: comparing COPD patients with healthy volunteers and laboratory model with activities of daily living
Source: BMC Pulm Med. 2013 Apr 27;13:27. doi: 10.1186/1471-2466-13-27 (PMC3663820; doi:10.1186/1471-2466-13-27)
Supplement: Additional file 7 — Interindividual variability of anxiety ratings (A2) following moderate and high level stimulus exposure by subject type (Healthy and COPD). Note that the range of ratings at matched A1 is similar between subject type. [file 1471-2466-13-27-S7.doc]

**Additional File 7**

Interindividual variability of anxiety ratings (A2) following moderate and

high level stimulus exposure by subject type (Healthy and COPD).

Note that the range of ratings at matched A1 is similar between subject type.

Anxiety Rating

(%FS)

100%

0%

Moderate Discomfort (A1 = 60%) High Discomfort (A1 = 90%)

Healthy COPD

Healthy COPD
